# Induction of altered gene expression profiles in cultured bovine granulosa cells at high cell density

**DOI:** 10.1186/s12958-016-0221-6

**Published:** 2017-01-05

**Authors:** Anja Baufeld, Dirk Koczan, Jens Vanselow

**Affiliations:** 1 Institute of Reproductive Biology, Leibniz Institute for Farm Animal Biology (FBN), Wilhelm-Stahl-Allee 2, 18196 Dummerstorf, Germany; 2 Institute for Immunology, University of Rostock, 18055 Rostock, Germany

**Keywords:** Bovine, Granulosa cells, Cell density, Gene expression, Signaling pathways, Microarray, Marker genes

## Abstract

**Background:**

In previous studies it has been shown that bovine granulosa cells (GC) cultured at a high plating density dramatically change their physiological and molecular characteristics, thus resembling an early stage of luteinization. During the present study, these specific effects on the GC transcriptome were comprehensively analysed to clarify the underlying mechanisms.

**Methods:**

GC were cultured in serum free medium with FSH and IGF-1 stimulation at different initial plating density. The estradiol and progesterone production was determined by radioimmunoassays and the gene expression profiles were analysed by mRNA microarray analysis after 9 days. The data were statistically analysed and the abundance of selected, differentially expressed transcripts was re-evaluated by qPCR. Bioinformatic pathway analysis of density affected transcripts was done using Ingenuity Pathway Analysis.

**Results:**

The data showed that at high plating density the expression of 1510 annotated genes, represented by 1575 transcript clusters, showed highly altered expression levels. Nearly two-thirds were up- and one third down-regulated. Within the top up-regulated genes *VNN2*, *RGS2* and *PTX3* could be identified, as well as *HBA* or *LOXL2*. Down-regulated genes included important key genes of folliculogenesis like *CYP19A1* and *FSHR*. Ingenuity pathway analysis identified “AMPK signaling” as well as “cAMP-mediated signaling” as major pathways affected by the alteration of the expression profile. Main putative upstream regulators were *TGFB1* and *VEGF*, thus indicating a connection with cell differentiation and angiogenesis. A detailed cluster analysis revealed one single cluster that was highly associated with the upstream regulator beta-estradiol. Within this cluster key genes of steroid biosynthesis were not included, but instead, other genes importantly involved in follicular development, like *OXT* and *VEGFA* as well as the three most down-regulated genes *TXNIP*, *PAG11* and *ARRDC4* were identified.

**Conclusions:**

From these data we hypothesize that high density conditions induce a stage of differentiation in cultured GC that is similar to early post-LH conditions in vivo. Furthermore we hypothesize that specific cell-cell-interactions led to this differentiation including transformations necessary to promote angiogenesis.

**Electronic supplementary material:**

The online version of this article (doi:10.1186/s12958-016-0221-6) contains supplementary material, which is available to authorized users.

## Background

During folliculogenesis the pre-ovulatory LH surge triggers ovulation and induces the transformation of the estradiol-producing follicle into the progesterone-producing corpus luteum. This massive reorganization of morphological and physiological aspects of the two somatic cell layers, granulosa and theca, is accompanied by well-defined alterations of the gene expression profiles [1, 2]. Cell culture models are an important tool to elucidate the underlying molecular mechanisms and pathways. In a previous study we could show that cultured bovine granulosa cells (GC) characteristically change the expression of specific marker transcripts under high density culture conditions, thus possibly mimicking an early stage of luteinization [3]. As observed in vivo, but triggered by the pre-ovulatory LH-surge, genes involved in steroid biosynthesis such as *CYP11A1*, *CYP19A1* and *HSD3B1* were down-regulated as well as transcripts encoding the gonadotropin receptors *FSHR* and *LHCGR*. In addition the expression of genes encoding the cell cycle regulator cyclin D2, *CCND2*, or the proliferation cell nuclear antigen, *PCNA*, was also down-regulated. Conversely, *VNN2*, *RGS2* and *PTGS2*, encoding vanin-2 (vascular non-inflammatory molecule 2), the regulator of G protein signaling and the key enzyme for prostaglandin synthesis cyclooxygenase-2 showed an up-regulation as observed in vivo after LH stimulation [4–8]. Besides these drastic changes in the gene expression profiles, the follicle cell layers convert into the physiologically and morphologically different corpus luteum (CL) after ovulation. The main function of the CL is the progesterone production to establish and maintain an oncoming pregnancy [9, 10]. First steps of this differentiation process occur shortly after the LH surge modulating the gene expression of key enzymes of steroidogenesis [11]. Apart from LH, growth factors as well as cytokines are known to be associated with the regulation of ovulation and luteal function [12, 13]. For a proper function of the CL a highly developed vascular system is essential, highlighting the importance of angiogenesis, which is involved in follicular and CL development [12, 14–16]. From this point of view a profound change of angiogenic factors should also be visible in the altered gene expression profile of cultured GC, suggestively mimicking the process of early luteinization. To address this question we performed a genome-wide transcriptome analysis using the previously described long-term GC culture model of increasing plating density [3, 17]. The production of the steroid hormones estradiol (E2) and progesterone (P) was analysed in addition to the characterization of the gene expression profiles of the cells under normal and high density conditions. We expect that a detailed knowledge of molecular changes induced under high density conditions in bovine GC would be a prerequisite to further analyse the relevance of this in vitro observation for the in vivo situation. In order to validate the used in vitro model, the data were compared with a previous in vivo transcriptome analysis studying effects of the pre-ovulatory LH surge on the transcriptome of theca and granulosa cells [6].

## Methods

### Tissue collection and cell culture

Ovaries were collected from a local abattoir and transported in cold 1x PBS containing penicillin (100 IU), streptomycin (0.1 mg/ml) and amphotericin (0.5 μg/μl). Follicular fluid with loosely attached or free floating granulosa cells were collected by aspiration with a syringe and 18 G needle from small to medium sized follicles (<6 mm) and collected in 1x PBS (with antibiotics). By this isolation procedure it is possible to obtain nearly pure granulosa cells without contaminating theca cells [4]. Living cells were counted in a hemocytometer using the trypan blue exclusion method and cryo-preserved in fetal calf serum containing 10% DMSO (Roth, Karlsruhe, Germany). Granulosa cell preparations were cell pools collected from 15 to 30 follicles per ovary of 30 to 50 ovaries, meaning that pools from at least 15 different cows with a non-defined cyclicity status were included in the replicates. Culture plates were coated shortly before the onset of culture with collagen R (0.02%; Serva, Heidelberg, Germany) to improve the attachment of cells to the surface [3]. Cells were cultured serum-free in α-MEM containing L-Glutamin (2 mM), sodium bicarbonate (0.084%), BSA (0.1%), HEPES (20 mM), sodium selenite (4 ng/ml), transferrin (5 μg/ml), insulin (10 ng/ml), non-essential amino acids (1 mM), penicillin (100 IU/ml) and streptomycin (0.1 mg/ml). For optimal culture conditions and the re-initiation of *CYP19A1* gene expression FSH at 20 ng/ml (Sigma Aldrich, Steinheim, Germany), R^3^ IGF-1 at 50 ng/ml (Sigma Aldrich), and androstenedione at 2 μM (Sigma Aldrich) were supplemented to the media. The cells were either plated at normal density of 1.0x10^5^ cells/well or at high density of 10.0x10^5^ cells/well in 24 well plates. All reagents were purchased from Biochrom AG (Berlin, Germany) if not stated otherwise. GC were maintained for 9 days at 37 °C and 5% CO_2_. Culture media were replaced every 2 days. In previous studies and according to our preliminary results it has been shown that after a rapid decline following dissociation and culturing (data not shown) E2 production and the expression of *CYP19A1*, the key gene of E2 biosynthesis, are re-initiated under long term culture conditions in GC thus partly mimicking a pre-LH stage of follicular differentiation [3, 18, 19].

### Determination of E2 and P4 concentrations

Progesterone concentrations were determined using an optimized direct competitive ^3^H-radioimmunoassay (RIA) [3, 4, 20]. The tracer, [1,2,6,7-3H(N)] progesterone, was purchased from PerkinElmer (Boston, USA) and the rabbit-raised antibody was purified by chromatography. The measurement was performed in a liquid scintillation counter (LSC) with an integrated a RIA-calculation programme (TriCarb 2900 TR, PerkinElmer). The intra- and interassay coefficients of variation (CVs) were 7.6% and 9.8%, respectively. The detection limit was 7 pg/ml. Media were diluted 1:40 in RIA-buffer and measured in duplicates. The concentration of estradiol was determined using a modified competitive ^3^H-RIA with the tracer [2,4,6,7-3H] estradiol-17β (GE Healthcare, Freiburg, Germany). The intra- and interassay CVs were 6.9% and 9.9%, respectively. The detection limit of the E2-RIA was 3 pg/ml. The analysis was done with undiluted media in duplicates. All measurements (ng/ml) were expressed relative to the extracted amount of RNA (ng) per cell preparation to normalize for cell numbers assuming a constant RNA amount per cell.

### RNA preparation and cDNA synthesis

Isolation of total RNA was done with the NucleoSpin^®^ RNA Kit (Macherey-Nagel, Düren, Germany) following the manufacturer’s protocol. RNA concentration was measured with a NanoDrop 1000 Spectrophotometer (Thermo Scientific, Bonn, Germany). cDNA synthesis was performed with MMLV reverse transcriptase (GeneOn, Ludwigshafen, Germany) using oligo-(dT) primers (2 ng/μl) and random hexamer primers (4 ng/μl, both Roche, Mannheim, Germany). The cDNA was cleaned using the High Pure Purification Kit (Roche) and diluted in 50 μl of the provided elution buffer.

### Quantitative Real-Time PCR

Gene expression analysis was done by quantitative real-time PCR with SensiFast™ SYBR No-ROX (Bioline, Luckenwalde, Germany) and gene-specific primers (listed in Additional file 1: Table S1). For the following reaction 0.25 and 0.5 μl cDNA were amplified in a total volume of 12 μl and the values of both were averaged considering different dilutions. The reaction was quantified in a LightCycler^®^ 480 instrument (Roche) with ensuing cycle conditions: pre-incubation at 95 °C for 5 min, 40 amplification cycles of denaturation at 95 °C for 20 s, annealing at 60 °C for 15 s, extension at 72 °C for 15 s, and a single-point fluorescence acquisition for 10 s. Melting point analysis was done immediately afterwards to ensure the amplification of the correct products. The length of each PCR product was checked by agarose gel electrophoresis (3%, ethidium bromide stained). Cloned PCR products, which were sequenced before for authentication, were co-amplified as external standards. Of these, dilutions were freshly prepared to obtain five different concentrations of standards (5 x 10^−12^-5 x 10^−16^ g DNA/reaction). qPCR values were normalized to the reference gene *RPLP0*, which showed very similar expression values under low and high density culture conditions in contrast to *RPS18* and *B2M* (Additional file 1: Table S2).

### Microarray profiling and statistics

Microarray analysis was performed with RNA from cultured bovine GC plated at two different cell densities. RNA was processed from *n* = 6 (3 samples per group) GC preparations as described above and quality was checked in a Bioanalyzer Instrument (Agilent Technologies, St. Clara, CA, USA). Amplification, labelling and hybridization to the Bovine Gene 1.0 ST Array was accomplished according to the supplier’s instructions using the “GeneChip® Expression 3’Amplification One-Cycle Target Labeling and Control Reagents” (Affymetrix, St. Clara, CA, USA). Samples were hybridized overnight in the GeneChipR Hybridization Oven (Affymetrix) and visualized using the Affymetrix GeneChip Scanner 3000. The original data were further processed using the Expression Console (V1.3.1.187; Affymetrix). Normalization, background reduction and gene-level summary was performed using the Robust Multichip Average (RMA) procedure with default settings. Principal component analysis was done with the Software Expression Console using default settings. Array results have been uploaded to the GEO database (GSE79311). Further comparative analysis of the data was realized with the Transcriptome Analysis Console 3.0 (TAC3.0, Affymetrix) using the Analysis of Variance (ANOVA) integrated in the software. The false discovery rate (FDR) procedure was also implemented in TAC3.0 using the Benjamini-Hochberg model [21]. Levels of significance were set with (fold change) │FC│ of >1.5, *p* < 0.05 and FDR < 0.05. For hierarchical clustering default settings of TAC3.0 are used, where the distance is the Euclidean distance and is computed by the complete linkage method. All additional statistics were performed using SigmaPlot 12.0 Statistical Analysis System (Jandel Scientific, San Rafael, CA, USA). The Pearson Product Moment procedure was used for correlation analysis.

### Ingenuity Pathway Analysis (IPA)

Bioinformatic pathway analysis was done with the Ingenuity Pathway Analysis tool (IPA, Qiagen, Hilden). For this, the generated list of differentially expressed transcripts according to the defined threshold values of FC, p-value and FDR (see above) was applied to the analysis tool. From these 1575 differentially expressed transcript clusters of the Bovine Gene 1.0 ST Array 1346 could be mapped by IPA to specific pathways, functions and upstream regulators.

## Results

### Expression profiling of GC cultured at different cell densities

**Fig. 1:**
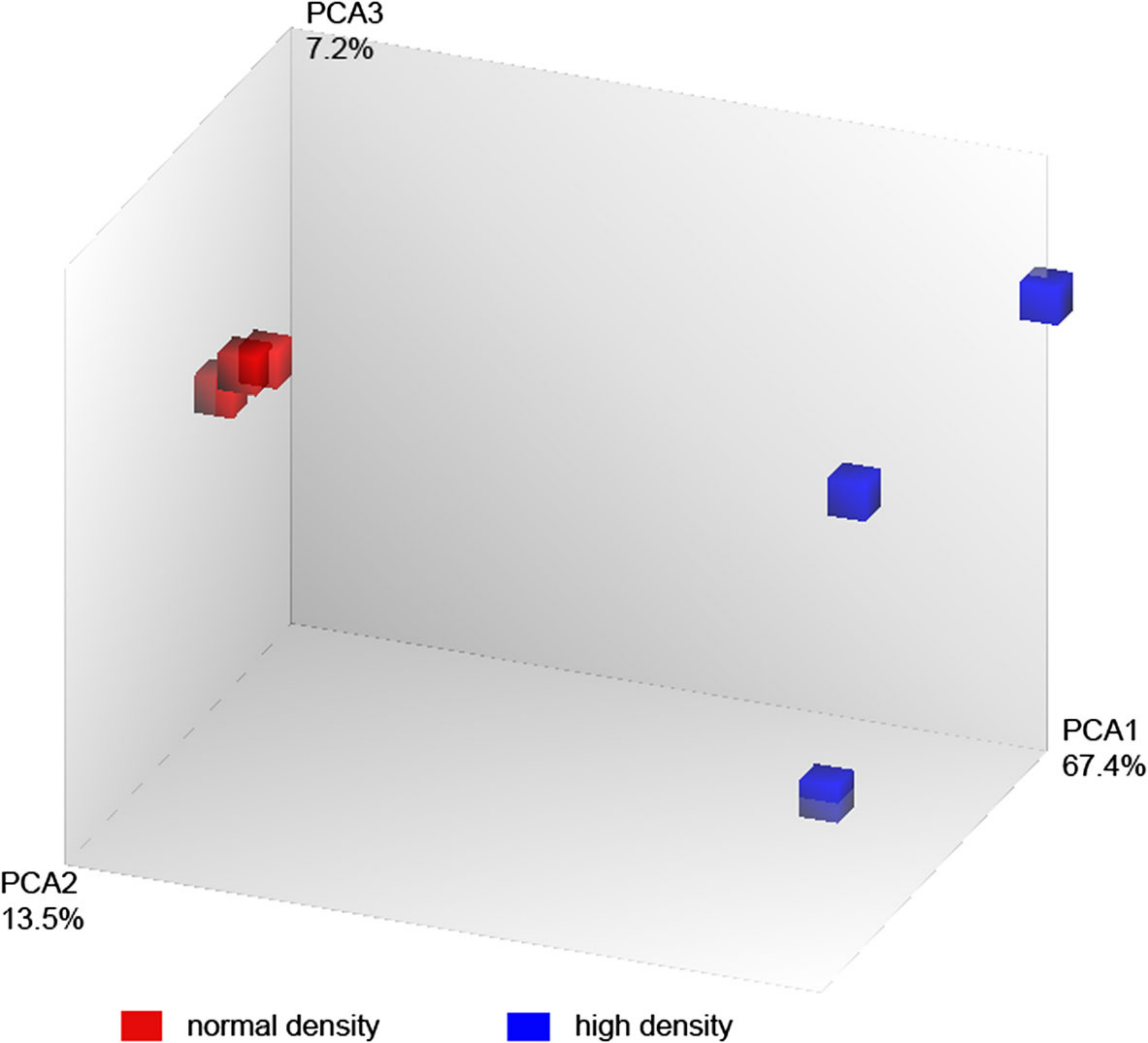
Principal component analysis (PCA) capturing differences in the transcriptome of cultured GC at different densities. Each symbol represents one sample, thus revealing the most significant variance between the different cell culture conditions which are indicated in red for the normal density or blue for the high density

As a first approach the mRNA microarray data were subjected to principal component analysis (PCA) to reduce the multidimensionality of datasets and to identify principal components with the highest variation. By this, individual samples can be plotted to estimate similarities and differences and to display the variance between datasets [22]. In the present analysis, each axis is assigned as a percentage reflecting the fraction of total variation (88.2%). This analysis revealed greatest variability on the x-axis with a variation of 67.4% (PCA1, Fig. 1). Here a clear separation of the GC cultured at normal (red) or high density (blue) is reflected. The gene expression levels were tightly clustered in GC cultured at normal density (red), but to a much lesser degree at high cell density (blue). This could be observed in the second most significant variation of the y-axis. But the observed variance of 13.5% (PCA2, Fig. 1) was much lower than that of PCA1.

The Bovine Gene 1.0 ST Array Chip includes nearly 200,000 probe sets, representing 26,288 transcript clusters. Of these, 1575 clusters (=1510 annotated genes) were found significantly different (│FC│ > 1.5; *p* < 0.05; FDR < 0.05) in the high density versus the normal density cultures (Additional file 1: Table S3). 669 clusters were down-regulated, whereas 906 showed up-regulation. Within the 669 down-regulated clusters only 42 displayed FC ≤ −3. Among these *CYP19A1*, *FSHR* and *INHA* could be detected as highly affected genes (Table 1). Additionally, an exceptional down-regulation of genes involved in glucose metabolism and oxidative stress like *TXNIP* (thioredoxin interacting protein; FC −79.5), *ARRDC4* (arrestin domain-containing 4; FC −8.1) or xanthine dehydrogenase (*XDH*; FC −5.2) could be observed. Also the pregnancy-associated glycoprotein 11 (*PAG11*; FC −15.5) was highly down-regulated. *PAG11* was previously shown to be expressed in bovine cumulus cells [23]. Furthermore, genes involved in cell-cell signaling or cell-matrix interactions are found to be down-regulated, e.g. *NRG1* (FC −4.9) and *SRGN* (FC −4.1), coding for neuregulin 1 and the proteoglycan serglycin, respectively. A relatively large number of genes or probe sets (146) revealed remarkable up-regulation (FC ≥ 3), including the previously described inflammatory genes *VNN2* and *PTX3*, or the regulator of G-protein signaling, *RGS2* (Additional file 1: Table S3). In addition, genes involved in extra-cellular-membrane (ECM) crosslinking and structure were up-regulated, e.g. keratins (*KRT18* and *KRT8*) as well as lysyl oxidases (*LOX*; *LOXL2; LOXL4*). Lysyl oxidases are also known to be connected to hypoxia as well as the genes *HBA* (FC 53.5), coding for hemoglobin alpha 2 and *EGLN3* (FC 12.8), coding for a hypoxia-inducible factor 3 of the egl-9 family (Table 2).

**Table 1:** Twenty top down-regulated genes in high density vs. normal density GC culture

Transcript cluster ID	Gene symbol	Gene title	FC
12837074	*TXNIP*	thioredoxin interacting protein	−79.50
12832821	*PAG11*	pregnancy-associated glycoprotein 11	−15.47
12793470	*ARRDC4*	arrestin domain containing 4	−8.13
12688063	*CYP19A1*	cytochrome P450, family 19, subfamily A, polypeptide 1	−7.02
12836111	*PIK3R3*	phosphoinositide-3-kinase, regulatory subunit 3 (gamma)	−6.55
12850069	*LOC512293*	G2/mitotic-specific cyclin-B1-like	−5.49
12703781	*XDH*	xanthine dehydrogenase	−5.36
12769776	*SLC43A2*	solute carrier family 43, member 2	−4.91
12823470	*NRG1*	neuregulin 1	−4.87
12739111	*SUSD4*	sushi domain containing 4	−4.83
12893122	*ASPN*	asporin	−4.77
12703820	*FSHR*	follicle stimulating hormone receptor	−4.64
12780359	*DHRS9*	dehydrogenase/reductase (SDR family) member 9	−4.59
12774405	*INHA*	inhibin, alpha	−4.53
12726330	*ANO3*	anoctamin 3	−4.20
12826119	*SRGN*	serglycin	−4.09
12892568	*OMD*	osteomodulin	−4.06
12678550	*SST*	somatostatin	−3.90
12871419	*RASL11B*	RAS-like, family 11, member B	−3.78
12685216	*KCNAB1*	potassium voltage-gated channel, shaker-related subfamily, beta member 1	−3.77

*FC*, fold change; *P* < 0.05; FDR < 0.05

**Table 2:** Twenty top up-regulated genes in GC under high density vs. normal density culture conditions

Transcript cluster ID	Gene symbol	Gene title	FC
12812382	*HBA*	hemoglobin, alpha 2	53.48
12683283	*AHSG*	alpha-2-HS-glycoprotein	36.57
12894529	*LOXL2*	lysyl oxidase-like 2	19.01
12718011	*TGM2*	transglutaminase 2 (C polypeptide, protein-glutamine-gamma-glutamyltransferase)	17.07
12864766	*KRT18*	keratin 18	16.63
12803191	*NRN1*	neuritin 1	13.97
12854172	*UPP1*	uridine phosphorylase 1	13.77
12793341	*EGLN3*	egl nine homolog 3 (C. elegans)	12.82
12893064	*IL33*	interleukin 33	12.59
12856851	*KRT8*	keratin 8	12.02
12889017	*LPL*	lipoprotein lipase	9.74
12830373	*CDKN1C*	cyclin-dependent kinase inhibitor 1C (p57, Kip2)	9.63
12688416	*CHAC1*	ChaC, cation transport regulator homolog 1 (E. coli)	9.56
12698536	*IL1RN*	interleukin 1 receptor antagonist	8.75
12817678	*SYT17*	synaptotagmin XVII	8.37
12860539	*SLC38A4*	solute carrier family 38, member 4	7.67
12748152	*CLDN5*	claudin 5	7.05
12703992	*PTGES*	prostaglandin E synthase	7.05
12876752	*LOX*	lysyl oxidase	7.04
12721639	*NCALD*	neurocalcin delta	6.84

*FC* fold change; *P* < 0.05; FDR < 0.05

**Fig. 2:**
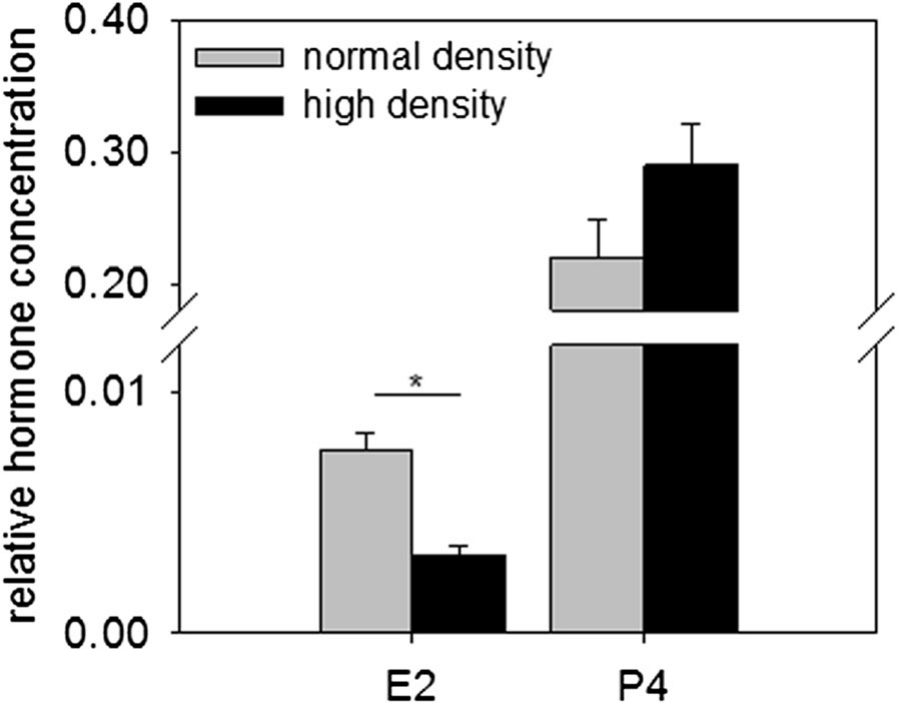
Hormone concentrations in GC cultured at different plating densities. Estradiol (E2) concentrations significantly decreased when GC were cultured at high cell density (*black bars*) compared to cells at normal density (*grey bars*). On the other hand the progesterone (P4) concentration tended to increase at high cell density. Hormone concentrations (ng/ml) are normalized to total RNA amounts (ng) of cell preparations to correct for cell numbers; mean values and SEMs are shown (*n* = 3, *P* < 0.05, t-test)

Although hypoxic conditions are likely to occur apoptotic processes seem rather inhibited than promoted by high plating density. This is suggested by the significant up-regulation of the anti-apoptotic genes *BCL2* (FC 2.0) and *BCL3* (FC 1.7) in accordance with the down-regulation of pro-apoptotic transcripts *CASP4* (FC −2.6) and *CASP8* (FC −1.7; Additional file 1: Table S3). This might be explained by positive effects of more intense cell-cell contacts on cell survival in this primary cell culture model. The analysis of hormone concentrations showed that E2 was significantly lower and P4 tended to higher concentrations under high plating density conditions (Fig. 2).

### Re-evaluation of microarray data by qPCR and identification of genes regulated in vivo by LH and in vitro by plating density

**Fig. 3:**
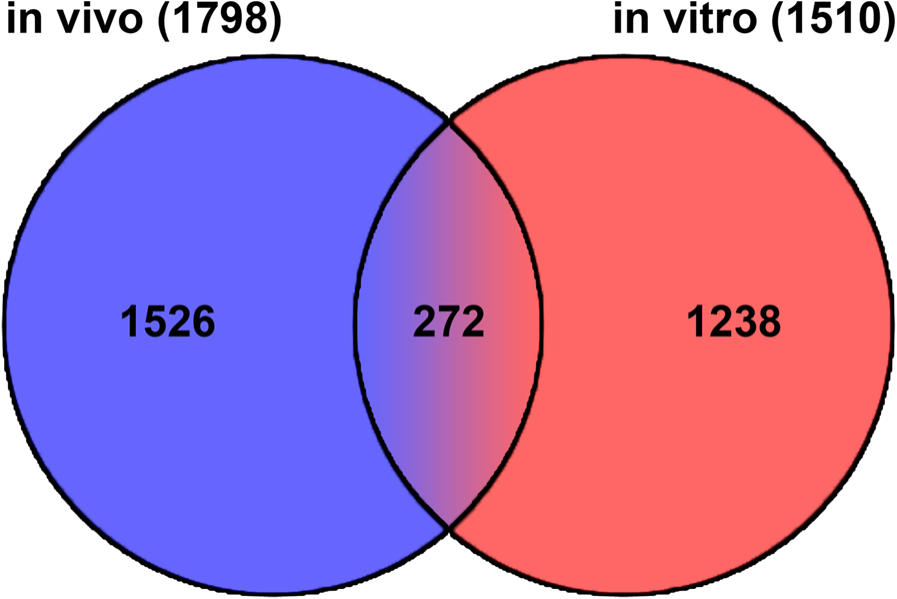
Numbers of genes regulated by high density in vitro and by LH in vivo. Total numbers of regulated genes are shown in brackets. In vivo data are derived from Christenson et al. [6]

Transcript levels of selected key genes of folliculogenesis were re-analysed by qPCR. Considering the transcript abundance levels as determined by qPCR and microarrays the Pearson product moment correlation analysis showed significant (*p* < 0.05) correlations for all analysed genes with coefficients between 0.78 and 0.99 (Table 3). Highest correlation coefficients could be observed for the down-regulated genes *CYP19A1* and *FSHR* as well as for the up-regulated *RGS2* and *VNN2.* Comparing data from a former in vivo Microarray analysis with the present in vitro experiments 272 genes were found significantly regulated in both studies (Fig. 3 and Additional file 1: Table S4). Of these, 143 were down-regulated and 129 up-regulated in vitro under high density conditions. Not all of the listed genes were regulated in the same manner. Instead, 22.7% of the genes were contrarily regulated (Table 4). Nevertheless, besides established genes that are strongly regulated during luteinization (e.g. *CYP19A1*, *FSHR*, *RGS2*) also other genes not yet known to be involved in granulosa cell differentiation were highly regulated in vivo as well as in our in vitro model and thus can likewise be considered as marker genes of early luteinization, e.g. *ITPKA* (inositol-triphosphate 3-kinase A), *SRGN* (serglycin) and *AHSG* (alpha-2-HS-glycoprotein). For nearly all genes shown in Table 4 a high and significant correlation between the in vivo and in vitro microarray study could be observed.

**Table 3:** Comparison of qPCR and microarray data from GC cultured under high vs. normal density culture conditions

Gene symbol	Gene name	FC qRT-PCR	FC microarray	Correlation coefficient
*CYP19A1*	cytochrome P450, family 19, subfamily A, polypeptide 1, aromatase	−11.09	−7.02	0.99
*CYP11A1*	cytochrome P450, family 11, subfamily A, polypeptide 1	1.65	1.24^a^	0.98
*HSD3B1*	hydroxy-delta-5-steroid dehydrogenase, 3 beta- and steroid delta	−2.43	−1.2^a^	0.98
*FSHR*	follicle stimulating hormone receptor	−5.97	−4.64	0.99
*LHCGR*	luteinizing hormone/choriogonadotropin receptor	−4.12	−2.35	0.98
*PTGS2*	prostaglandin-endoperoxide synthase 2 (prostaglandin G/H synthase)	1.09^a^	1.27^a^	0.78
*RGS2*	regulator of G-protein signaling 2	4.84	3.3	0.99
*VNN2*	vanin 2	7.75	5.88	0.99
*PTX3*	pentraxin 3, long	3.95	3.02	0.96
*PCNA*	proliferating cell nuclear antigen	−1.16^a^	−1.18^a^	0.83
*CCND2*	cyclin D2	−1.69	−1.37^a^	0.96

*FC* fold change, *qRT*-*PCR* was normalized to the reference gene *RPLP0*; microarray data was normalized with the RMA method; all correlations were significant with *P* < 0.05; genes labelled with ^a^were not classified as significant according to microarray analysis, because the FC did not reach the threshold of 1.5 or −1.5

**Table 4:** Comparison of microarray data from GC cultured under high vs. normal density conditions in vitro and before and after the pre-ovulatory LH surge in vivo

Gene symbol	Gene title	FC in vitro	FC in vivo	corr. *r*	*p*-value
*P4HA3*	prolyl 4-hydroxylase, alpha polypeptide III	1.7	75.8	0.997	0.00001
*ITGA4*	integrin, alpha 4 (antigen CD49D, alpha 4 subunit of VLA-4 receptor)	1.9	7.2	0.995	0.00004
*WWC2*	WW and C2 domain containing 2	1.5	3.9	0.995	0.00004
*TACC3*	transforming, acidic coiled-coil containing protein 3	−1.7	−5.4	0.995	0.00004
*ATAD2*	ATPase family, AAA domain containing 2	−1.8	−8.1	0.994	0.00005
*INHBA*	inhibin, beta A	−2.4	−29.5	0.994	0.00005
*CAPG*	capping protein (actin filament), gelsolin-like	1.7	7.3	0.994	0.00005
*LOC784007*	uncharacterized LOC784007; SLAM family member 9-like	−2.0	−21.8	0.993	0.00007
*ITPKA*	inositol-trisphosphate 3-kinase A	2.2	6.7	0.993	0.00007
*QSOX1*	quiescin Q6 sulfhydryl oxidase 1	1.6	5.4	0.992	0.00009
*NDRG1*	N-myc downstream regulated 1	4.3	63.8	0.992	0.00010
*SAT1*	spermidine/spermine N1-acetyltransferase 1	1.6	15.0	0.991	0.00013
*CDCA3*	cell division cycle associated 3	−1.6	−11.0	0.989	0.00017
*RGS2*	regulator of G-protein signaling 2, 24 kDa	3.3	194.0	0.989	0.00020
*HEG1*	HEG homolog 1 (zebrafish)	−2.1	−29.7	0.985	0.00035
*PTX3*	pentraxin 3, long	3.0	643.9	0.958	0.00257
*SRGN*	serglycin	−4.1	−29.9	0.924	0.00841
*ADAMTS1*	ADAM metallopeptidase with thrombospondin type 1 motif, 1	1.9	28.5	0.967	0.00166
*CYP19A1*	cytochrome P450, family 19, subfamily A, polypeptide 1	−7.0	−397.6	0.912	0.01140
*HSD11B1*	hydroxysteroid (11-beta) dehydrogenase 1	2.4	14.5	0.898	0.01493
*FSHR*	follicle stimulating hormone receptor	−4.6	−4.1	0.896	0.01555
*LHCGR*	luteinizing hormone/choriogonadotropin receptor	−2.4	−7.9	0.811	0.05005
*AHSG*	alpha-2-HS-glycoprotein	36.6	152.3	0.809	0.05110
*VNN2*	vanin 2	5.9	116.0	0.749	0.08659
*CDKN1C*	cyclin-dependent kinase inhibitor 1C (p57, Kip2)	9.6	24.4	0.718	0.10777

*FC* fold change; corr, correlation, calculated by Pearson Product Moment analysis

### Pathway analysis and upstream regulators

Potentially affected pathways under high density culture conditions were analysed using the IPA tool. The differentially expressed genes referred to 64 “Canonical Pathways” (Table 5 and Additional file 1: Table S5). “AMPK Signaling” (AMP-activated protein kinase) was highly affected including 30 differentially regulated genes. The z-score indicated an inactivation of this pathway. “cAMP-mediated signaling” was another pathway affected by high density culture conditions and was predicted to be activated (z-score 1.257). Thirty two differentially expressed genes could be connected to this pathway including the gonadotropin receptors *FSHR* and *LHCGR* (Additional file 1: Table S5). The IPA tool also revealed a high number of upstream regulators, which could be involved in the altered gene expression profiles under high density culture conditions (Additional file 1: Table S6). Top regulators are *TGFB1* (transforming growth factor, beta 1), *VEGF* (vascular endothelial growth factor), *TP53* (tumor protein p53) and β-estradiol with 245, 103, 214 and 231 differentially regulated target genes, respectively. For these regulators (except *VEGF*) activation was predicted indicating a higher activity under the high density culture conditions. The predicted upstream regulator *TGFB1* was significantly up-regulated itself with a fold change of 3.7, thus clearly suggesting a substantial role of this growth factor during density associated alterations. The top cellular and molecular functions assigned by IPA included “cellular assembly and organization” thus highlighting increasing effects of cell-cell interactions under high density culture conditions (Table 6). This observation is also in accordance with the observation that genes involved in cell-cell or cell-matrix interactions were significantly regulated.

**Table 5:** Top 20 canonical pathways identified by IPA

Ingenuity canonical pathways	-log(*p*-value)	*p*-value	Ratio	z-score^a^	Number of affected molecules	Total number of molecules^b^
AMPK Signaling	4.00	0.0001	0.183	−0.471	30	164
Hepatic Fibrosis/Hepatic Stellate Cell Activation	3.76	0.0002	0.180		29	161
Acetate Conversion to Acetyl-CoA	3.55	0.0003	0.800		4	5
Ovarian Cancer Signaling	3.45	0.0004	0.190		23	121
cAMP-mediated signaling	3.25	0.0006	0.162	1.257	32	197
Mitotic Roles of Polo-Like Kinase	3.08	0.0008	0.236		13	55
Glioma Signaling	3.04	0.0009	0.198	0.258	18	91
Cell Cycle Control of Chromosomal Replication	2.87	0.0013	0.308		8	26
Xenobiotic Metabolism Signaling	2.79	0.0016	0.151		33	218
LXR/RXR Activation	2.69	0.0020	0.181	0.775	19	105
Calcium Signaling	2.62	0.0024	0.161	1.698	25	155
Eumelanin Biosynthesis	2.59	0.0026	0.750		3	4
FXR/RXR Activation	2.50	0.0032	0.174		19	109
ATM Signaling	2.50	0.0032	0.214	−1.265	12	56
Ethanol Degradation IV	2.46	0.0035	0.333		6	18
LPS/IL-1 Mediated Inhibition of RXR Function	2.45	0.0035	0.151	0.378	28	185
Glioma Invasiveness Signaling	2.43	0.0037	0.211	0.577	12	57
GADD45 Signaling	2.33	0.0047	0.316		6	19
Superpathway of Cholesterol Biosynthesis	2.31	0.0049	0.280		7	25
Oxidative Ethanol Degradation III	2.26	0.0055	0.357		5	14

^a^z-score reflects activation, if values are positive and inactivation, if values are negative

^b^Total number of molecules present on the Bovine Gene 1.0 ST Array that are assigned to specific canonical pathways by IPA

**Table 6:** Top 10 Molecular functions assigned by IPA

Category	*p*-value^a^	Number of molecules affected	Total number of molecules^b^
Cellular Growth and Proliferation	3.8E-20 - 8.8E-05	563	5452
Cell Death and Survival	2.47E-15 - 9.69E-05	483	4545
Cell Cycle	6.45E-15 - 7.83E-05	232	2255
Cellular Assembly and Organization	6.45E-15 - 7.34E-05	260	4244
DNA Replication, Recombination, and Repair	6.45E-15 - 8.49E-05	128	1979
Cellular Movement	1.01E-13 - 8.74E-05	331	3043
Cellular Development	3.58E-12 - 8.8E-05	528	5390
Lipid Metabolism	4.8E-12 - 7.98E-05	238	2105
Small Molecule Biochemistry	4.8E-12 - 7.98E-05	258	4000
Cell Morphology	4.52E-11 - 9.41E-05	376	4268

^a^ *P*-value range is according to different subcategories of the molecular functions assigned by IPA

^b^The total number of molecules present on the Bovine Gene 1.0 ST Array that are assigned to specific molecular functions by IPA

### Single cluster analysis

**Fig. 4:**
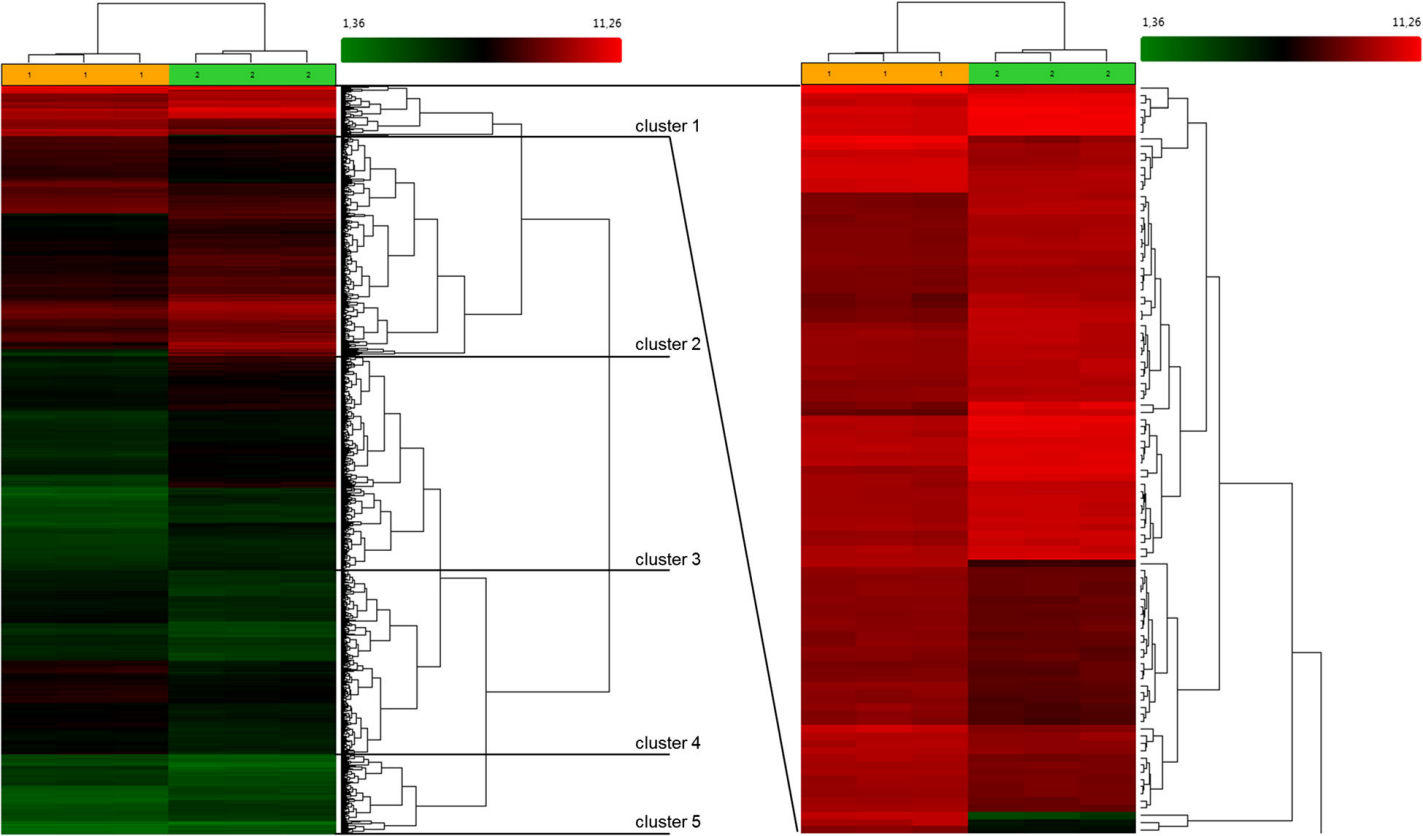
Hierarchical clustering and heatmap of regulated genes in high versus normal density GC culture. The different culture conditions are shown as orange and green above the heatmap reflecting the normal density culture and high density culture samples, respectively. The heatmap visualizes the signal for every gene in all 3 samples of each culture condition from lower hybridization signals (*green*) to higher signals (*red*)

Hierarchical clustering of the microarray data revealed a very clear separation of individual samples collected from GC cultures under normal vs. high density conditions (Fig. 4). To obtain a more detailed insight into the functional importance of similarly regulated genes, one cluster was analysed with the IPA tool. The whole gene dendrogram was divided into 5 clusters (Fig. 4, *left panel*). In this analysis “cluster 1”, which included 104 genes (Additional file 1: Table S7), turned out to be the most interesting one including the three most down-regulated genes *TXNIP* (FC −79.5), *PAG11* (FC −15.47) and *ARRDC4* (FC −8.14) at the bottom of the heat map (Fig. 4, *right panel*). One important upstream regulator identified by IPA was β-estradiol (Additional file 1: Table S8). Interestingly, no genes coding for key enzymes of steroid biosynthesis are clustered here. But still other commonly known genes involved in folliculogenesis can be found, e.g. the significantly up-regulated genes *OXT*, coding for oxytocin (FC 1.6) and *VEGFA*, coding for the vascular endothelial growth receptor A (FC 2.1) as well as down-regulated genes *INHBA* (inhibin beta A, FC −2.4) and FST (follistatin, FC −1.8).

## Discussion

### High plating density of cultured GC induces specific alterations of the gene expression profile

Principal component analysis as well as hierarchical clustering revealed a clear separation of samples cultured under normal compared to high density conditions. This clearly indicates that increasing cell plating density of bovine GC led to genome-wide and specific alterations of the gene expression profiles. On the other hand, however, it was also obvious that the samples cultured at high density showed greater variability among each other compared to those under normal culture conditions. So far we have no conclusive explanation for this observation, but nevertheless, the separation of samples under normal and high density culture conditions was assigned to the highest variance by PCA according to their respective expression profiles. This is in line with previous studies, which revealed a change of physiological and molecular properties of GC cultured at increased cell densities [3, 17]. This was further confirmed by the steroid hormone profiles of GC cultured at normal and high cell density. When GC were cultured at high cell density, the E2 concentration decreased, which is in accordance with the down-regulation of *CYP19A1* expression, coding for the key enzyme of estradiol synthesis. The concentration of P4 on the other hand tended to increase as it is known in vivo after the LH surge [24]. In previous studies, where effects of plating density have been reported in cultured bovine and ovine granulosa cells, the analyses were restricted to selected aspects as steroidogenesis and angiogenesis [25, 26]. To our knowledge our explorative study is the first one analysing effects of increased cell density using a whole genome approach in any cell type. The data can be used for further in depth studies on selected candidate genes with independently collected samples. Accurate a-priori-calculations of the required sample size are now possible on the basis of the now known effect sizes (= fold change) of individual genes.

Several of the regulated genes that could be identified in high density cultures had been also found in previous in vivo studies focusing on genes affected by the pre-ovulatory LH surge [4, 6, 11, 27]. All together nearly 58% of the genes, which were regulated by increasing the cell density in vitro were determined as up-regulated thus suggesting that increased density induced a differentiation process in GC with an intense activation of specific key genes. Among them we found inflammation-related genes, e.g. *VNN2*, *PTX3* and *ADAMTS1.* These genes have also been shown up-regulated in vivo by LH, thus suggesting a functional role during the folliculo-luteal transition. *PTX3* has been shown to be important in ECM remodelling within the follicle leading to infertility in *PTX3* ^*−/−*^ mice [28]. Interestingly, several genes which are involved in ECM modulation and structure were found affected in high density cultures. Keratins as well as lysyl oxidases were significantly up-regulated thus indicating involvement of cell to cell interactions. Remarkably, lysyl oxidases are also known to be connected to hypoxia [29–31]. Transcripts of *HIF1A*, however, have not been found elevated in our bovine GC culture model, in contrast to a recently published study using ovine cells [26]. Possibly, this could be due to different culture models in particular regarding the selected duration of cell culture. In our study, cells were cultured for 9 days to enable re-initiation of *CYP19A1* expression and E2 production, whereas the ovine cells were analysed after 2 to 3 days in culture. Density induced regulation on the post-translational level due to hydroxylation of HIF1A, however, cannot be excluded. This mechanism has been shown in previous studies [32–34]. The up-regulation of other hypoxia-related genes (*HBA* and *EGLN3*), however, suggest that hypoxic conditions occur in GC cultures under high density conditions, presumably within the observed tight cell clusters described in Baufeld et al. [3]. Studies by others revealed that the induction of hypoxic factors is necessary for the ongoing differentiation process in the follicle [35, 36]. However, it is still unclear whether hypoxic conditions, in particular those presumably caused by increasing cell density in the GC layer of dominant follicles, are in fact essential signals during the folliculo-luteal transition in vivo [37].

Besides previously described marker genes of folliculogenesis, extensively down-regulated genes were *TXNIP* and *ARRDC4. TXNIP* has been described as a redox-sensitive signaling protein with a connection to the glucose metabolism [38]. A direct interaction between glucose and the thioredoxin-interacting protein has been described in liver and muscle cells, whereby low *TXNIP* levels can improve the glucose uptake [39, 40]. The resulting higher intracellular concentration of glucose could in turn lead to an increased expression and promotor activity of *TXNIP* [41]. Having this in mind, the massive down-regulation of *TXNIP* in GC cultured at high density suggests a higher uptake and consumption of glucose under high density conditions. This is in line with the observation of a higher glucose consumption of in vitro grown murine follicles after hCG administration [42]. For *ARRDC4* a similar function could be hypothesized, because *ARRDC4* and *TXNIP* belong to the same protein family of alpha-arrestin and have similar effects on glucose metabolism [43, 44]. Another highly regulated gene is *NRG1*, encoding neuregulin 1, which is a cell-cell signaling protein with at least 15 different isoforms resulting in a wide variety of biological functions during embryonic development and postnatally [45]. In the ovary, its regulation seems to be highly dynamic. Directly after hCG treatment *NRG1* expression was found induced [46, 47]. Another study showed a decreased expression of *NRG1* after 12 h [48]. We could identify a significant down-regulation of *NRG1* in high density GC cultures, which might mimic the long term LH effects. Interestingly, also the expression of *SRGN*, encoding the ECM proteoglycan serglycin, was found down-regulated under high density conditions thus resembling the LH-induced regulation of this ECM modulator during the late pre-ovulatory follicular phase [6, 49], where it may play a role for ECM modulation during the folliculo-luteal transition phase. Suggestively, a similar modulation of the ECM might be induced under high density conditions.

### Cell-cell communication pathways are affected in cultured GC under high density conditions

“AMPK Signaling” and “cAMP-mediated signaling” were identified as the top affected pathways, with the “AMPK signaling” predicted as inactivated under high density conditions. In a former study, LH-induced changes of AMPK phosphorylation have been shown in bovine luteal cells revealing an inactivation of AMPK by LH [50]. This is in line with our results thus suggesting that similar cell-cell interactions might be involved in the characteristic physiological and molecular alterations in cultured GC under high density conditions even in the absence of LH as a luteinizing agent. “cAMP-mediated signaling” could also be observed as highly influenced. The second messenger cAMP leads to an activation of different downstream targets. One of these targets could be identified as the protein kinase A (PKA) [51, 52]. Interestingly the PKA signaling cascade was described earlier to be involved in luteinization events in different species [53–55]. This is in accordance with the predicted activation of the cAMP-mediated signaling. Preliminary observations of our group, however, suggest that PKA signaling is not involved in the density induced alterations in vitro (unpublished data). Among the top upstream regulators identified by IPA was the transforming growth factor, *TGFB1*. The predicted activation is in good accordance with the observed up-regulation of this gene under high density conditions. A study in human granulosa cells demonstrated the inductive influence of *TGFB1* on cyclooxygenase-2 expression and prostaglandin E2 production [56], which is a reliable marker for approaching ovulation after the LH surge. *VEGF*, which is known to be influenced by *TGFB1* during angiogenesis [57], was identified as another main upstream regulator. Taken together the up-regulation of *TGFB1* and the identification of *VEGF* as another upstream regulator suggest that *TGFB1* initiates a phenotype in GC cultured at high cell density favouring angiogenesis, an essential prerequisite for luteinization. This is also supported by the observation that lysyl oxidases, which are important factors during angiogenesis [31, 58], are among the top up-regulated genes. Interestingly, in a recent study E2 was found to stimulate the expression of members of the lysyl oxidase family [59], having in mind that β-estradiol was identified as another upstream regulator. Lysyl oxidases are known to be responsible for the covalent crosslinking of collagen in the ECM [60, 61]. In regard to the observed up-regulation of a number of these genes (*LOX*, *LOXL1*, *LOXL2* and *LOXL4*) in the present study, increasing substrate-cell crosslinking of the cultured bovine GC can be hypothesized under high density conditions. “Cellular assembly and organization” has been identified as one of the most affected functions. This is in accordance with the observed regulation of genes involved in cell-cell or cell-matrix interactions. We identified a considerable number of genes involved in ECM remodelling as up-regulated with partly very high fold changes > 3. Here we found the keratins *KRT8* and *KRT18* thus implicating that a higher amount of these anchoring proteins is needed under high density conditions. These proteins are known as primary keratins and often form the filamentous network in the cytoplasm of cells [62] and are synthesized constantly [63, 64]. Besides the keratins, *PTX3* is known to be involved in remodelling the ECM during ovulation by binding hyaluronan to form a stable matrix in the cumulus oophorus [65]. *PTX3* ^*−/−*^ mice show severe defects in female fertility albeit having normal fertilization rates that led to the hypothesis that defects occur during cumulus expansion [65, 66].

### Hierarchical cluster analysis revealed co-regulation of genes by β-estradiol as a major upstream regulator

Hierarchical cluster analysis was performed to identify groups/clusters of genes with similar regulatory characteristics and thus possibly upstream effectors. Most interestingly, β-estradiol was identified as the major upstream regulator of the genes included in “cluster 1”. Genes encoding key enzymes of steroidogenesis were not present within this cluster, however, other genes essentially involved in folliculogenesis could be detected, e.g. follistatin (*FST*), inhibin beta A (*INHBA*) or oxytocin (*OXT*). Oxytocin, which showed an up-regulation in high density GC culture, is known to have a wide variety of functions. This peptide hormone induces physiological as well as behavioural changes [67, 68]. Previously, *OXT* could be established as a marker for luteinization showing an up-regulation at the time of ovulation [69–71]. It was further suggested that the peptide oxytocin influences steroidogenesis as a potent luteotropic factor in the corpus luteum [72, 73]. Interestingly, the hypothesis of cell-cell interactions is also discussed in connection with the up-regulation of progesterone by oxytocin [71]. The angiogenic gene *VEGFA* could also be identified within this cluster indicating a regulation by β-estradiol in general but also highlighting angiogenesis.

### In vitro high density conditions and the pre-ovulatory LH surge show similar effects on key genes of the folliculo-luteal transition

The microarray dataset could be largely validated by qPCR measurements of selected genes. In particular *CYP19A1*, *FSHR*, *RGS2* and *VNN2*, which have been previously suggested as marker genes for early luteinization [3, 4, 6–8, 74], showed a highly significant correlation comparing qPCR and microarray data. The analysis of effects of the pre-ovulatory LH-surge on antral granulosa cells revealed the regulation of 2741 genes [6]. The comparison between data of the present study and of this in vivo study revealed an interesting accordance of regulated genes. Both studies identified a common set of regulated genes, even though with partially huge differences in fold change values. This observation suggests that this specific set of genes might be also indirectly affected in vivo during the folliculo-luteal transition by increasing cell-cell interactions in the wake of the pre-ovulatory LH surge. Besides the formerly described highly affected genes *CYP19A1*, *FSHR*, *RGS2* or *VNN2* [3, 6, 8, 74, 75], also other interesting density as well as LH affected genes could be identified. Within this list there are genes, which have not yet been described as luteinization-associated markers in GC as *ITPKA* and *SRGN*, coding for the inositol-triphosphate 3-kinase A and serglycin. Serglycin acts as a regulator for proteolytic enzymes within the ECM further supporting the view that increasing density conditions in vitro reflect certain aspects of the LH-induced changes in vivo. ECM modifications within the pre-ovulatory follicle are certainly important processes during the folliculo-luteal transition [65]. Obviously, high density conditions in vitro and the LH surge in vivo exert similar signals on the granulosa cells thus suggesting that high density conditions in vitro drive the cultured cells towards a post LH stage of differentiation or early stage of luteinization, which is in line with our previous study [3]. However, part of these genes did not show the same direction of change. It is obvious that the high density granulosa cell culture model can mimic the LH surge induced transformations during folliculo-luteal transition phase only in parts. But nevertheless, the induced physiological alterations [3] and changes of the gene expression profiles clearly suggest that bovine granulosa cells cultured at different plating densities is an appropriate model to mimic certain aspects of the folliculo-luteal transition. This also raises the question, if the LH-induced transformation processes observed in vivo might be partly ascribed to indirect effects like enhanced cell-cell interactions by increasing cell density.

## Conclusions

During the present study effects of increased cell density were analysed for the first time by whole genome transcription profiling. The data revealed remarkable alterations of the gene expression profiles in GC under high density culture conditions favouring ECM remodelling and early angiogenesis. A subset of the affected genes has been previously identified in vivo as LH-regulated during the folliculo-luteal transition thus suggesting that increasing cell density can partially mimic processes of early GC luteinization even in the absence of LH. This is also supported by steroid hormone data showing reduced E2, but increased P4 production. Accordingly, we hypothesize that the fundamental alterations of gene expression profiles during the folliculo-luteal transition might not only be directly induced by LH, but in a subset of genes also indirectly by altered cell-cell interactions.

## Additional file

Additional file 1: The datasheets were named **Table S1**–**Table S8** containing additional information about the mRNA microarray analysis and subsequent Ingenuity Pathway Analysis. They are explicitly referenced within the text. (XLSX 267 kb)

## Abbreviations

*ADAMTS1*: A disintegrin and metalloproteinase with thrombospondin motifs 1
*AHSG*: Alpha-2-HS-glycoprotein
AMPK: AMP-activated protein kinase
*ARRDC4*: Arrestin domain containing 4
*B2M*: Beta-2-microglobulin
*BCL2*/*3*: B-cell lymphoma 2/3
BSA: Bovine serum albumin
cAMP: Cyclic AMP
*CASP4*/*8*: Caspase 4/8
*CCND2*: Cyclin D2
CL: Corpus luteum
*CYP11A1*: Cholesterol side-chain cleavage enzyme
*CYP19A1*: Aromatase
E2: Estradiol
ECM: Extracellular matrix
*EGLN3*: Hypoxia-inducible factor 3 of the egl-9 family
FC: Fold change
FDR: False discovery rate
FSH: Follicle stimulating hormone
*FSHR*: FSH receptor
*FST*: Follistatin
GC: Granulosa cells
*HBA*: Hemoglobin alpha 2
hCG: Human choriogonadotropin
*HIF1A*: Hypoxia-inducible factor 1-alpha
*HSD3B1*: Hydroxy-delta-5-steroid dehydrogenase
IGF-1: Insulin-like growth factor 1
*INHA*: Inhibin A
*INHBA*: Inhibin beta A
IPA: Ingenuity Pathway Analysis
*ITPKA*: Inositol-triphosphate 3-kinase A
*KRT8*/*18*: Keratin 8/18
LH: Luteinizing hormone
*LHCGR*: LH/choriogonadotropin receptor
*LOX*: Lysyl oxidase
*LOXL*: Lysyl oxidase like
LSC: Liquid scintillation counter
MEM: Minimal essential media
*NRG1*: neuregulin 1
*OXT*: Oxytocin
P4: Progesterone
*PAG11*: Pregnancy-associated glycoprotein 11
PBS: Phosphate buffered saline
PCA: Principal component analysis
*PCNA*: Proliferation cell nuclear antigen
PKA: Proteinkinase A
*PTGS2*: Prostaglandin-endoperoxidase synthase 2
*PTX3*: Pentraxin 3
qPCR: quantitative PCR
*RGS2*: Regulator of G protein signaling 2
RIA: Radioimmunoassay
RMA: Robust Multichip Average
*RPLP0*: Ribosomal protein, large subunit P0
*RPS18*: Ribosomal protein S18
*SRGN*: Serglycin
TAC: Transcriptome Analysis Console
*TGFB1*: Transforming growth factor beta 1
*TP53*: Tumor protein 53
*TXNIP*: Thioredoxin interacting protein
*VEGF(A)*: Vascular endothelial growth factor (A)
*VNN2*: Vanin 2
*XDH*: Xanthine dehydrogenase
